# Editorial: Advanced Immunization Technologies for Next Generation Vaccines

**DOI:** 10.3389/fimmu.2020.00878

**Published:** 2020-05-06

**Authors:** Donata Medaglini, Peter Andersen, Rino Rappuoli

**Affiliations:** ^1^Department of Medical Biotechnologies, University of Siena, Siena, Italy; ^2^Department of Infectious Disease Immunology, Statens Serum Institut, Copenhagen, Denmark; ^3^GSK, Siena, Italy; ^4^vAMRes Lab, Toscana Life Sciences, Siena, Italy; ^5^Imperial College, London, United Kingdom

**Keywords:** ADITEC, immunization technologies, vaccines, adjuvants, vaccine vectors, systems vaccinology, human immunology

The spectacular increase of life expectancy in many countries of the world is mainly due to the conquest of infectious diseases by vaccines, hygiene, and antibiotics. Vaccines have done and continue to do an excellent job in eliminating or reducing the impact of infectious diseases (1). Vaccines prevent 2.5 million deaths per year: every minute five lives are saved by vaccines worldwide. However, despite the huge progress made in past decades, there is still work to be done: for some important diseases we do not have a vaccine yet, and for others, currently available vaccines are not good enough. Thanks to the new technologies, vaccines can do much more for the needs of modern society. Several challenges and unmet needs still remain and require an urgent effort in vaccine research and development: emerging infectious diseases, infectious diseases linked to poverty, bacteria resistant to antibiotics, and non-communicable diseases.

To address these challenges new immunization technologies that allow for the development of safe and more effective vaccines are needed. New technologies and big data analysis, including genomics and systems biology, are fueling advances in our understanding of human immunology, transforming the old field of vaccinology, and shaping the future of medicine. Thanks to new technologies, vaccines have the potential to make an enormous contribution to the health of modern society by preventing and treating not only communicable diseases in all ages, but also non-communicable diseases such as cancer. The sophisticated science behind the development of modern vaccines has become so complex that scientists and policy-makers need to develop a new model for vaccine research (2–4), and joint research efforts that combine academic research with the vaccine development and manufacturing expertise found in commercial vaccine companies are essential.

To this aim, the high Impact Project ADITEC (Advanced Immunization Technologies; www.aditecproject.eu/) was funded by the 7th Framework Programme for Research and Innovation of the European Union (2). The project has contributed to accelerate the development of novel and powerful immunization technologies for the next generation of human vaccines. Scientists from 42 partner institutions in 13 different European countries and USA have joined forces in the ADITEC project. With a budget of about 30 million euros over 6 years, ADITEC has made significant advances in the development of novel immunization technologies, adjuvants, vectors and delivery systems, formulations, and vaccination methods optimized for different age groups and for different routes of administration. ADITEC has conducted 12 vaccine clinical trials and contributed to international regulation and standards for these novel technologies. Along with regularly setting up and running European training programmes, ADITEC has also created synergies and cross-fertilization between different research areas that have the potential to fill existing gaps and advance this knowledge well into the future. To date, ADITEC has a track record of over 325 scientific publications in international peer-reviewed journals (https://www.aditecproject.eu/publications/) and has generated innovations with high socio-economic impact (https://www.aditecproject.eu/category/reports/impact-reports/). New technologies have been developed that can effectively advance these innovations to the clinic and make a real difference for future health.

In this Research Topic are assembled a series of original research articles and reviews, which highlight the progresses made by the ADITEC project in the development of advanced immunization technologies.

Novel insights have been obtained in the field of adjuvants and delivery systems (Anderson et al.; Baldwin et al.; Christensen et al.; Obiero et al.; Olsen et al.; Santoro et al.; Tulli et al.; Vo et al.; Vono et al.), vectors (Gallinaro et al.; Nieuwenhuizen and Kaufmann), vaccine formulations (Billeskov et al.), delivery devices (deGroot et al.; Platteel, Nieuwenhuizen et al.), antigen design (Platteel, Liepe et al.), and prime–boost immunization strategies (Ciabattini, Pettini et al.). Together with structure-based design of immunogens this wealth of new information contribute to development of new immunization technologies with the potential to radically transform the field of vaccinology. Moreover, better infection models using clinically relevant influenza strains to test vaccine induced protection (Groves, McDonald et al.) have been developed and the potential impact of host microbiota on vaccine immune responses has been investigated [(5); Groves, Cuthbertson et al.].

Recent omics and systems biology big data platforms have yielded valuable insights and a systems vaccinology approach, integrating clinical, immunological, and *omics* data, has the potential to contribute to identification of markers that will guide the rational development of vaccines (6, 7). Here are reported the human molecular signatures of immunity and immunogenicity in infection and vaccination identified in the context of the ADITEC clinical studies and technologies developed to assess the human immune response at the level of the transcriptomic and proteomic response, T-cell and B-cell memory, cellular trafficking, and key molecular pathways of innate immunity, with an emphasis on mechanisms of protective immunity (Anderson et al.; Haks et al.).

Human challenge models, in which volunteers are experimentally infected with a pathogen of interest, offer the opportunity to identify both natural and vaccine-induced correlates of protection. In this Research Topic, we also highlight how the application of transcriptomics to human challenge studies allows for the identification of novel correlates and gives insight into the immunological pathways required to develop functional immunity (Barton et al.).

Vaccines need to be tailored for diverse age and target groups to optimally stimulate the host immune system. Indeed, the changes in the immune system of the elderly, where some immunological components are weakened (immunosenescence) while others such as inflammation are increased, are the basis of the reduced response to vaccination in the elderly (8). There is the need to design vaccine formulations, including vaccine adjuvants, that optimally stimulate the elderly immune system taking in consideration the need to avoid excess inflammation. Similarly, vaccination in infants, the other important target population of vaccination, face the challenge of an immature immune system that impose specific requirements on vaccines to be able to prime the necessary immune response in small children. In this issue are reported studies on the characterization of human vaccine immune responses in different age groups, including adults (Anderson et al.; Kratochvil et al.; Obiero et al.), elderly (Weinberger et al.), and children (Wilkins et al.), through the latest generation of advanced methodologies. The effect of age, gender, underlying disease, and medication in vaccine responses has also been investigated (Olafsdottir et al.).

With its broad portfolio of complementary expertise from both academia and the private sector integrated by a truly collaborative spirit, ADITEC represent a valuable model for future large integrated projects to tackle complicated problems for the benefit of society.

## Author Contributions

All authors conceived the outline of the manuscript. DM wrote the manuscript. RR and PA critically revised and approved the final version of the manuscript.

## Conflict of Interest

The authors declare that the research was conducted in the absence of any commercial or financial relationships that could be construed as a potential conflict of interest.
